# Supplementary material for: Unraveling MLL1-fusion leukemia: Epigenetic revelations from an iPS cell point mutation
Source: J Biol Chem. 2024 Sep 27;300(11):107825. doi: 10.1016/j.jbc.2024.107825 (PMC11541820; doi:10.1016/j.jbc.2024.107825)
Supplement: Supporting information [file mmc1.docx]

**Unraveling MLL1-fusion Leukemia: Epigenetic Revelations from an iPS Cell Point Mutation**

**Supporting Information**

Laila Kobrossy^a^, Weiyi Xu^b,c^, Chunling Zhang^d^, Wenyi Feng^a^, Christopher E. Turner^b^,

and Michael S. Cosgrove^a,^*

^a^Department of Biochemistry and Molecular Biology, SUNY Upstate Medical University, Syracuse, New York 13210, United States

^b^Department of Cell and Developmental Biology, SUNY Upstate Medical University, Syracuse, New York 13210, United States

^c^Current Address: Cell Biology Department, Harvard Medical School, Boston, MA

^d^ Department of Neuroscience and Physiology, SUNY Upstate Medical University, Syracuse, New York 13210, United States

**Running Title:** MLL1-mutant iPS cell changes suggest leukemia mechanism

**Key Words:** Epigenetic plasticity, H3K4 methylation, MLL1, SETd1A, EMT, WDR5, leukemic stem cell, iPS cells, precision medicine

*To whom correspondence should be addressed:

Michael S. Cosgrove, Ph.D.

SUNY Upstate Medical University

Department of Biochemistry and Molecular Biology

750 East Adams Street  
Syracuse, New York 13210

Email: [cosgrovm@upstate.edu](mailto:cosgrovm@upstate.edu)

Phone: (315) 464-7751

Supp. Fig. 1 and 2 are re-used from previous publications as indicated and are collected here to aid the reader in understanding the impact of the MLL1 R3765A substitution on the assembly and enzymatic activity of the MLL1 core complex *in vitro* and *in cellulo*.

**Supp. Fig. 1 |** **The MLL1 core complex predominately catalyzes H3K4 mono- and dimethylation.**

**Fig. S1 | MLL/SET1 family core complex single turnover kinetics. a)** Quantitative MALDI-TOF mass spectrometry showing methylation of an unmodified histone H3 peptide (aa. 1-20) after 24 hours for each human MLL/SET1 family core complex. **b)** reaction progress curves globally fitted to irreversible consecutive reaction models using DynaFit. Each timepoint is the mean percentage of total integrated area for each species in MALDI-TOF reactions. Error bars represent +/- S.D. from duplicate measurements. **c)** summary of rate constants derived from global fitting of reaction progress curves. This figure is re-used from Fig. 3 of reference ^5^.

**Supp. Fig. 1 |** **Cont.**


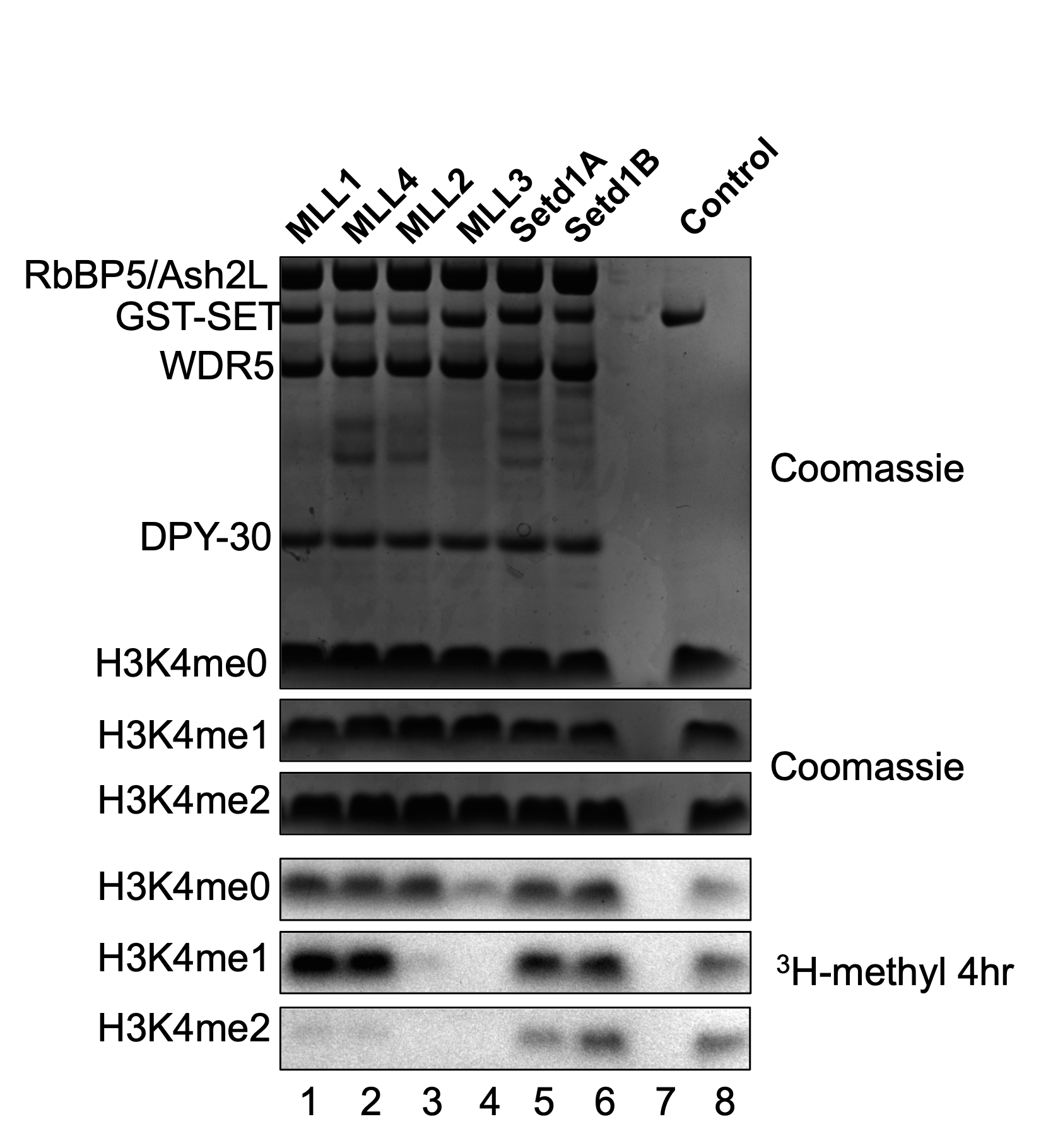


-10

-15

-60

-40

-10

-10

-10

-10

-10

kDa

**Fig. S1d | Biochemically reconstituted human MLL/SET1 family core complexes have different product specificities.** Each MLL/SET1 family SET domain in a fusion with GST was incubated with WRAD subunits, ^3^H-methyl SAM, and histone H3 peptide substrates that were unmodified (H3K4me0), previously monomethylated at H3K4 (H3K4me1) or previously dimethylated at H3K4 (H3K4me2). Upper panels show Coomassie-Blue stained SDS-PAGE gels, lower panels show ^3^H-methyl incorporation after 4 hours as shown by fluorography. The control lane shows the activity of the isolated MLL1 SET domain on 100 μM unmodified H3 peptide, which was included on each gel. This figure is re-used from Fig. 2c of reference ^5^.

**Supp. Fig. 2 | The R3765A substitution in MLL1 disrupts the assembly and enzymatic activity of the MLL1 core complex.**


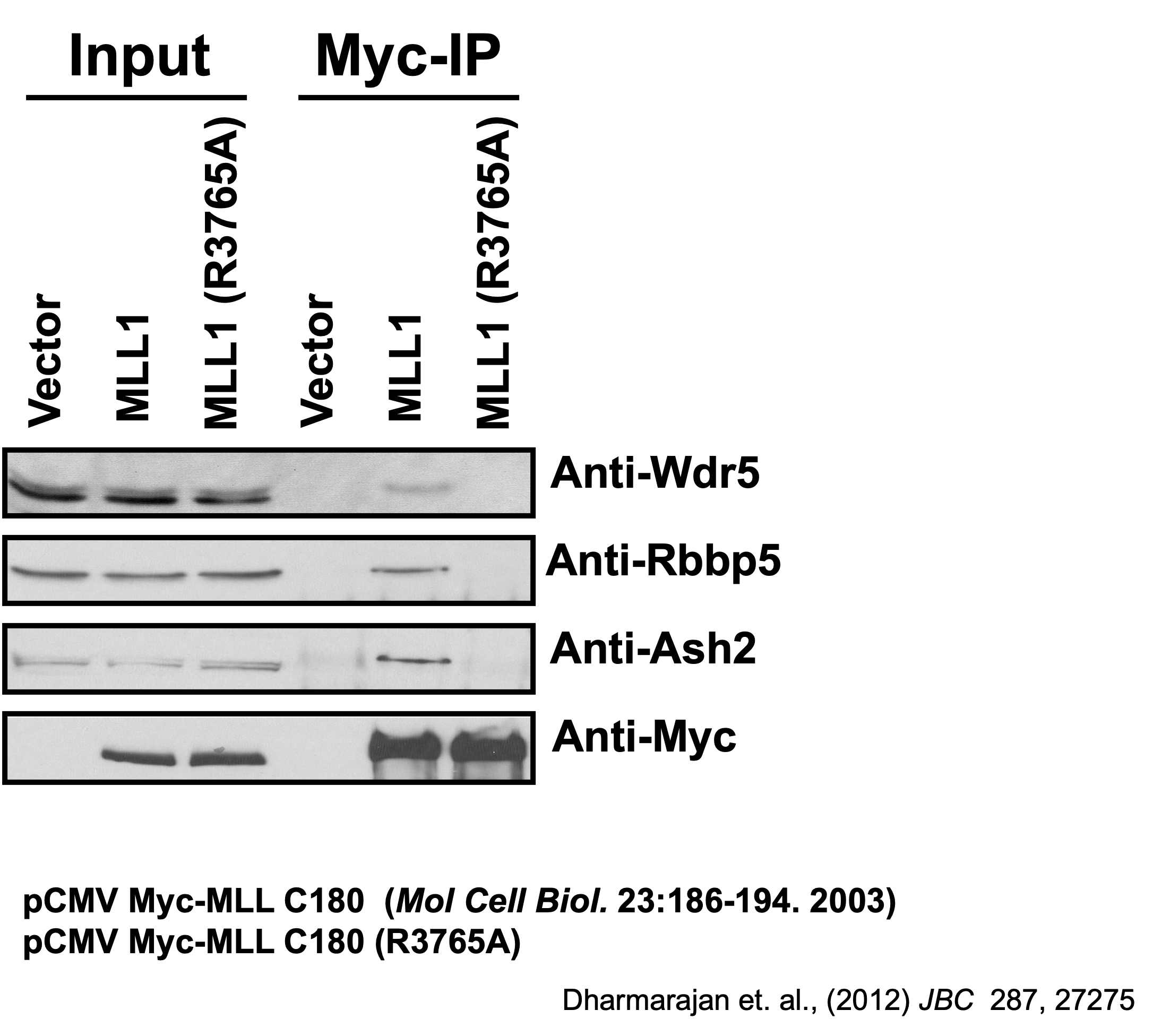


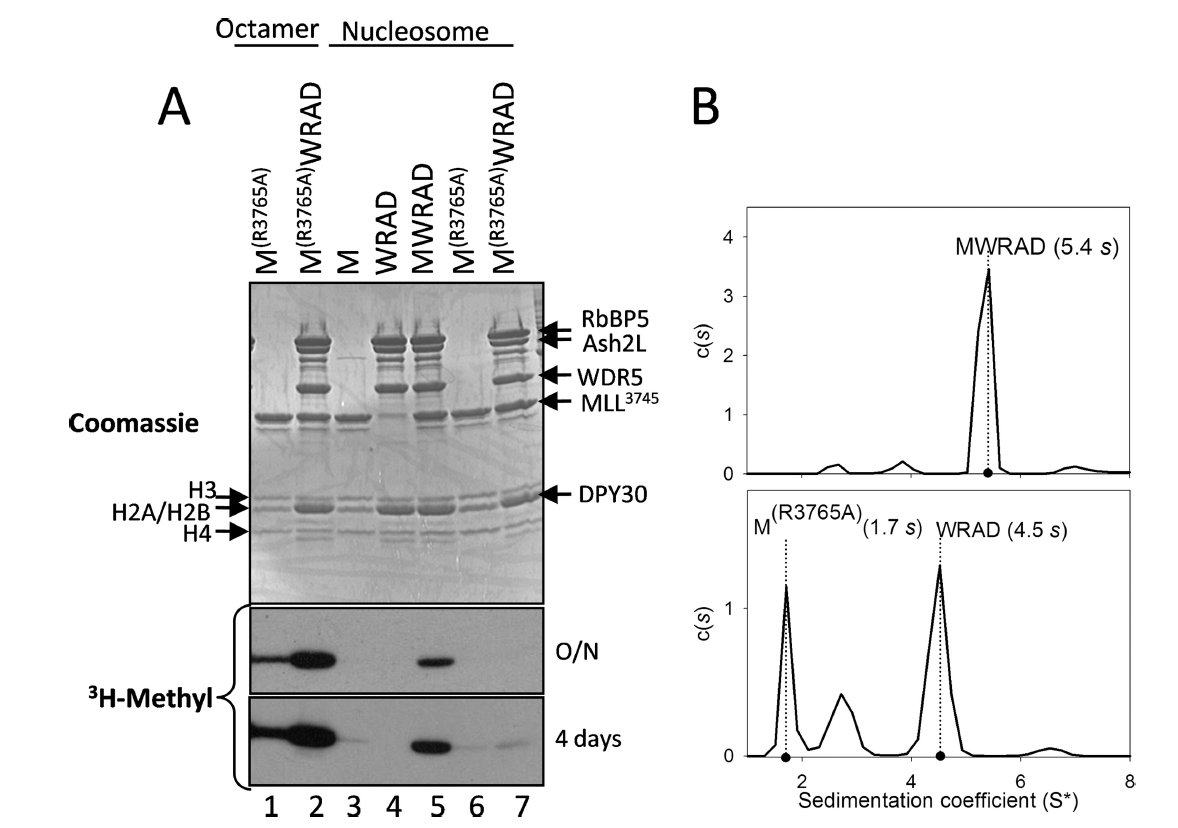


-15

-15

-15

kDa

**Fig. S2b |** **The MLL1 R3765A substitution abolishes methylation of nucleosome histone H3 by the MLL1 core complex.** Wild type (lane 5) or R3765A (lane 7) MLL1 variants were assembled with WRAD and incubated with ^3^H-AdoMet and a reconstituted nucleosomal substrate. Quenched reactions were separated by SDS-PAGE and stained with Coomassie (upper panel). ^3^H-methyl signal was visualized by fluorography (lower panels). This figure is re-used from Fig 6A of reference ^4^.

**Fig. S2a |** **The MLL1 R3765A substitution disrupts assembly of the MLL1 core complex in mammalian cells.** pCMV-Myc vectors encoding the 180 kDa C-terminal fragment of wild type or R3765A MLL1 proteins were transfected into HEK293 cells. Antibodies against c-Myc were used to immunoprecipitate each MLL1 variant. Pull downs were western blotted with anti-WDR5, anti-RbBP5 or anti-Ash2L antibodies. This figure is re-used from Fig. 2 of reference ^2^.

**Supp. Fig. 3 |**

**Fig. S3b |** Top 10 predicted CRISPR-Cas9 off-target loci identified by Applied Stem Cells. Sanger sequencing confirmed the integrity of all 10 loci in MT cells.


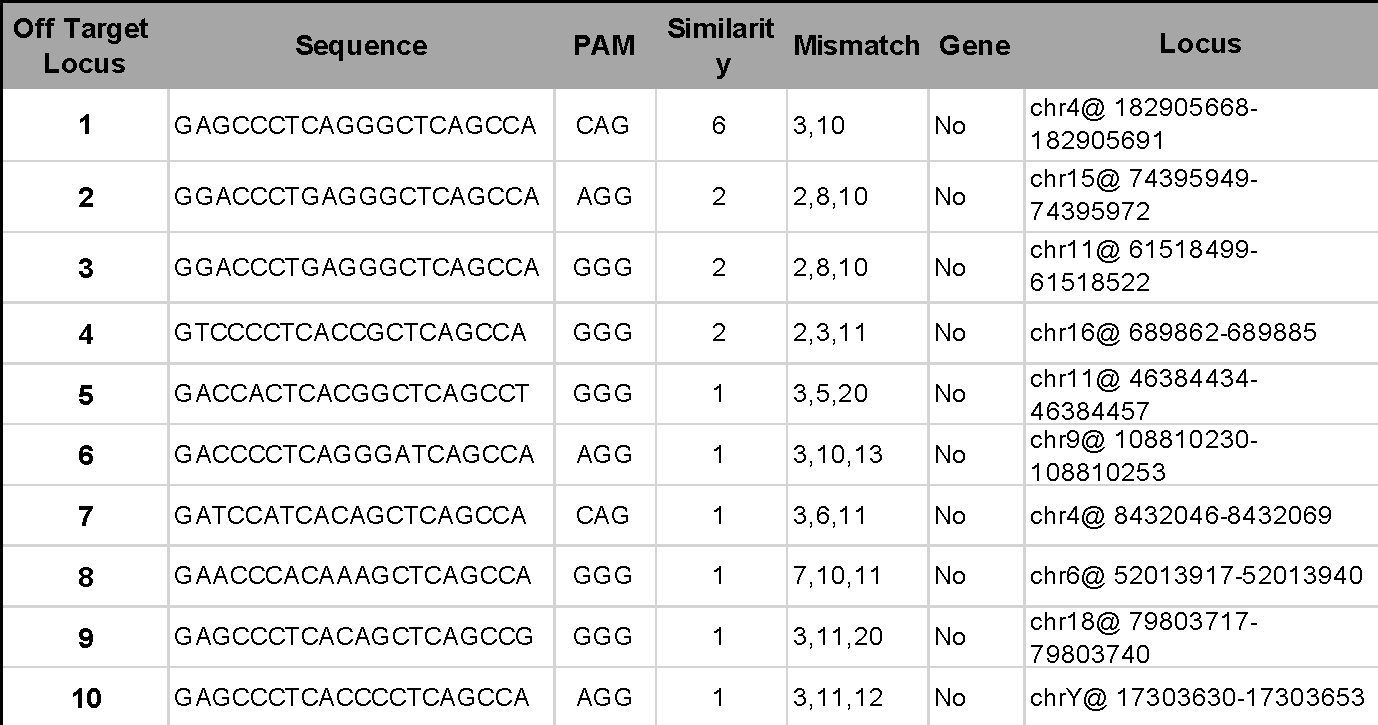

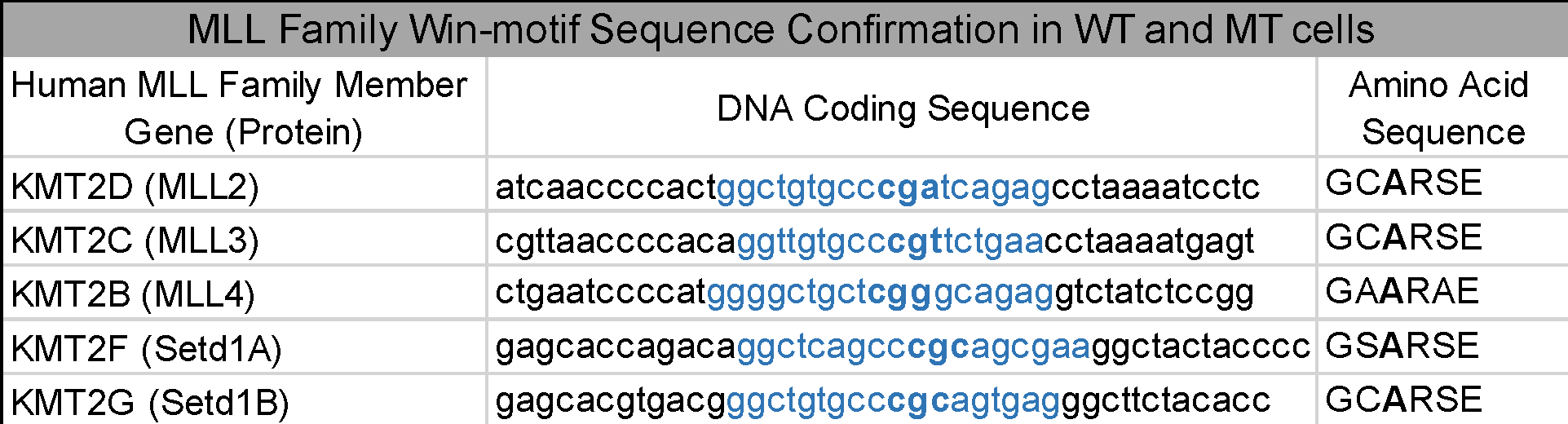


**Fig. S3a** **|** Integrity of all five Win-motif sequences for the other human MLL family members was confirmed in both WT and MT cells by Sanger sequencing. Win-motif coding sequence is in blue (key arginine coding sequences in bold).

**
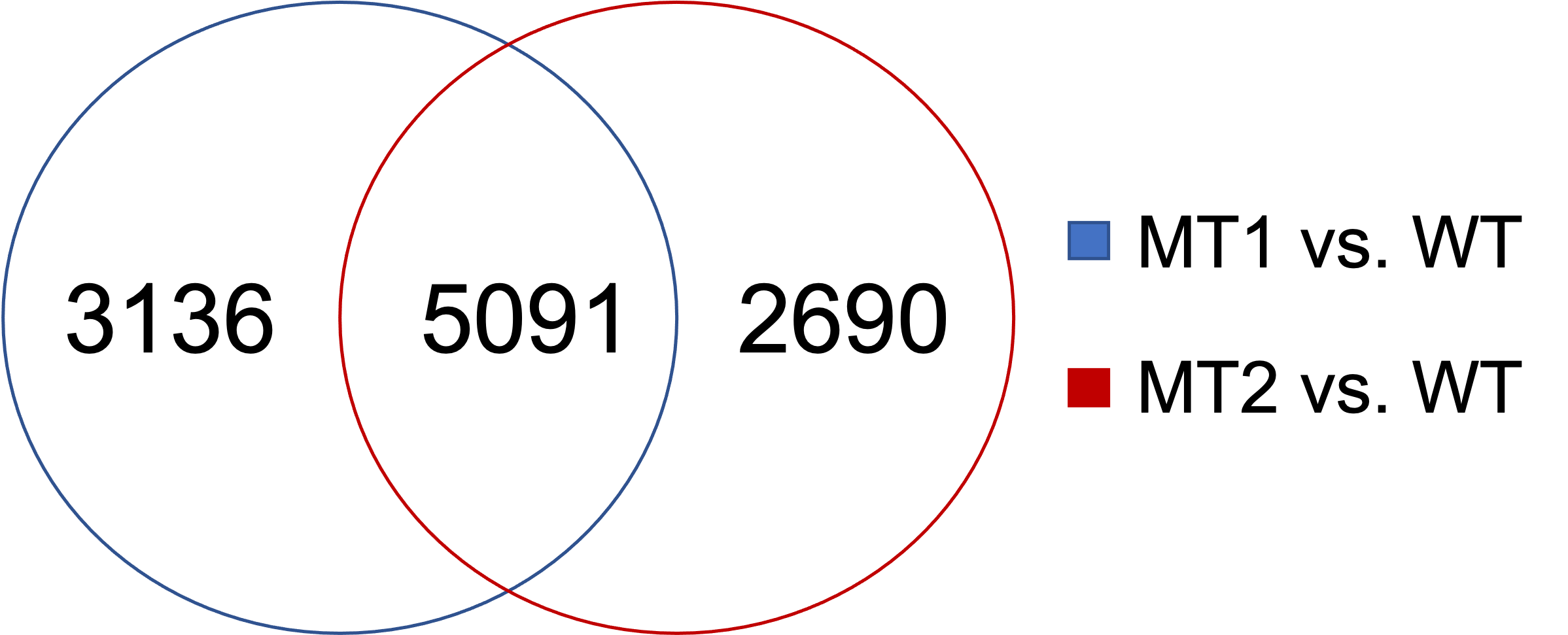

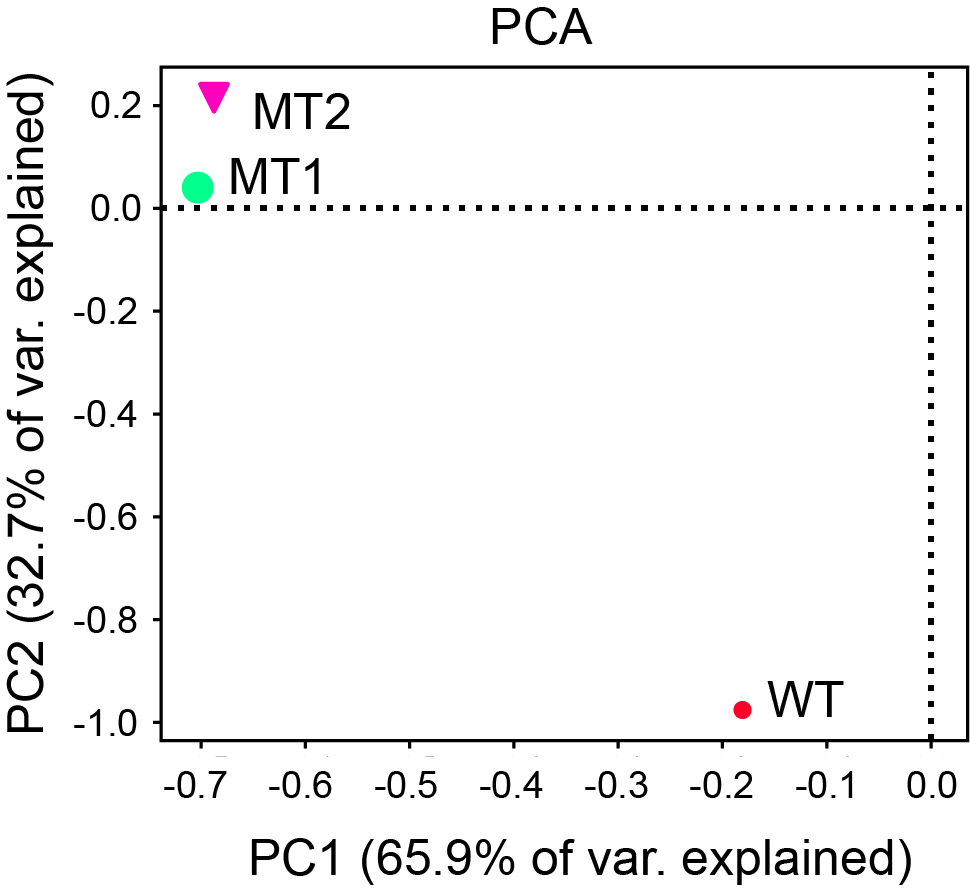
Supp. Fig. 3 |** **Cont.**

**Fig. S3d |** **Principal component analysis (PCA) of ChIP-seq results from Wild type and both MT IPS cell clones.** The grouping of both MT clones relative to WT confirms consistent changes in H3K4me3 ChIP-seq signals in the two MT clones.

**Fig. S3c |** Venn diagram showing the overlap of differential ChIP-Seq H3K4me3 peaks detected in MT1 and MT2 clones, analyzed relative to WT cells.

**Supp. Fig. 3 |** **Cont.**


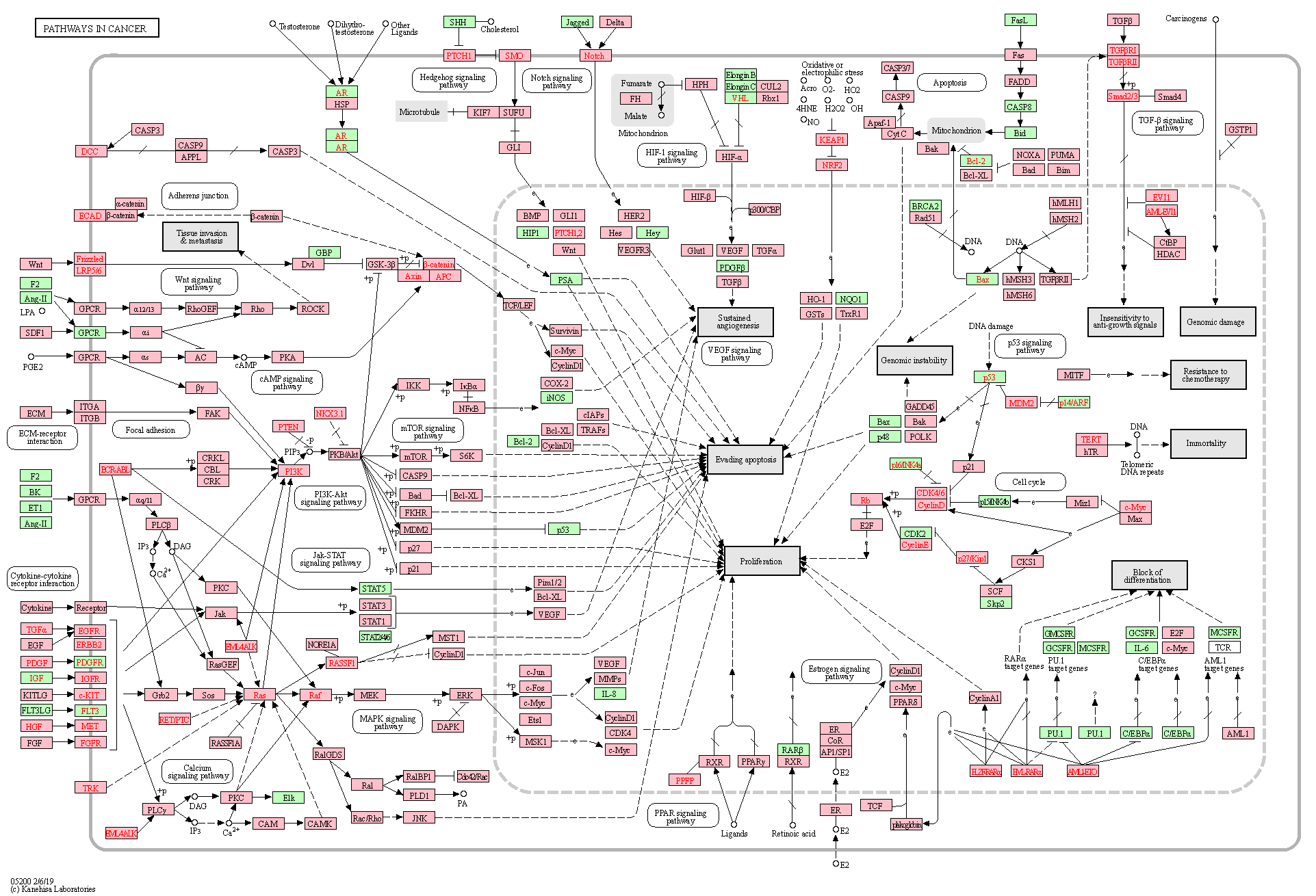


**Fig. S3e |** KEGG enrichment analysis of molecules involved in cancer pathways (pink) and represented in overlapping ChIP-Seq peaks in MT1 and MT2 clones normalized to WT cells.

**Supp. Fig. 4 |**


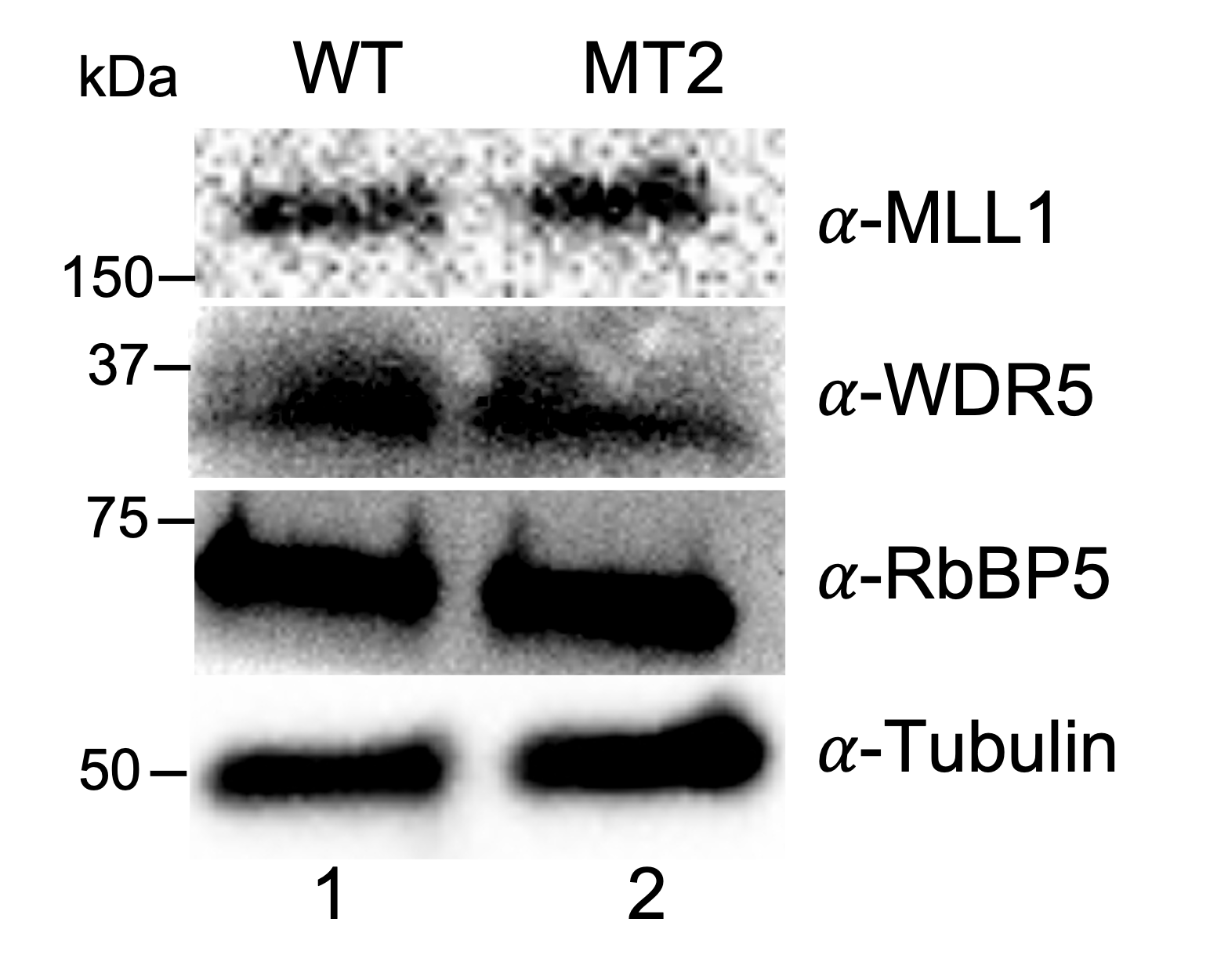


**Fig. S4b |** Protein levels of select MWRAD complex components in WT and MT2 cells. Whole cell extracts from and MT2 cells were immunoblotted using antibodies specific for MLL1, WDR5, and RbBP5. Tubulin was used as a loading control.


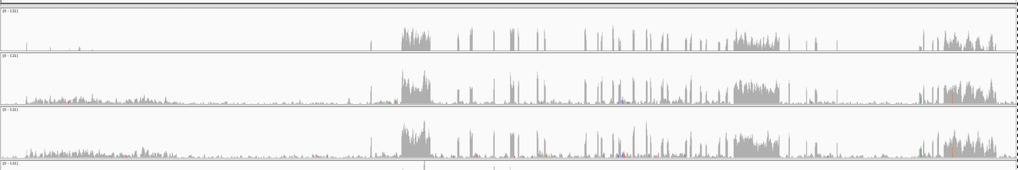

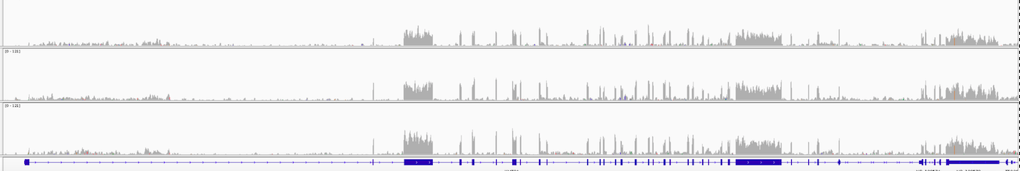

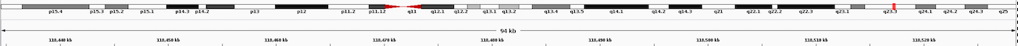


WT

MT

IGV: Human KMT2A gene locus

**Fig. S4a |** Integrated genomics view (IGV) of the human KMT2A locus in WT and MT (MT2) cells, showing identical transcript profile from multiple exons in triplicate RNA-seq samples.


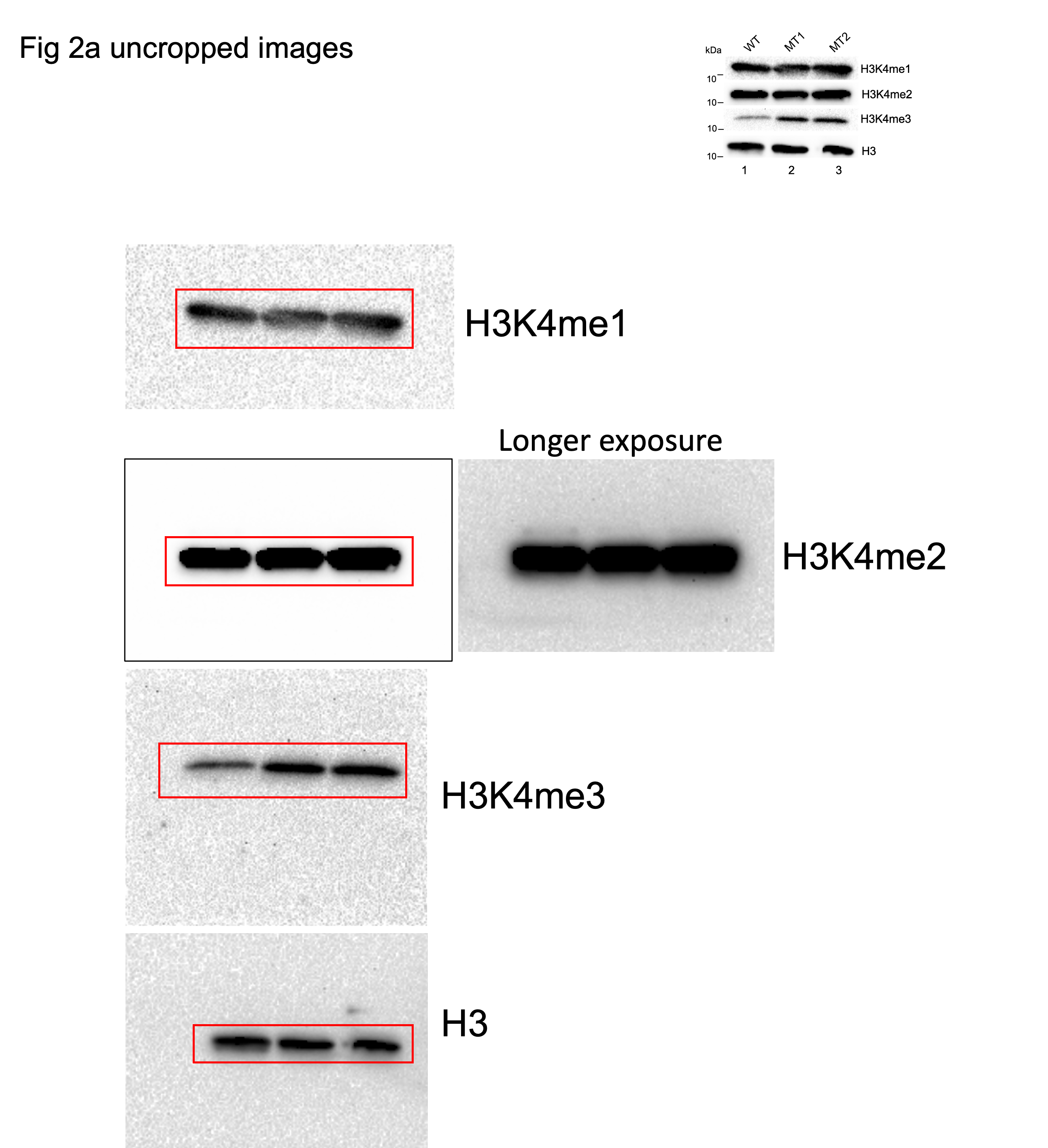


10-

10-

10-

10-

10-

kDa

**Uncropped Western Images for Figures 2A, 5C, 6A and S4B.**

A cropped version of each figure is shown for reference. In some cases, a longer exposure is shown to help visualize the blot outline. Cropped areas of each blot are outlined in red.


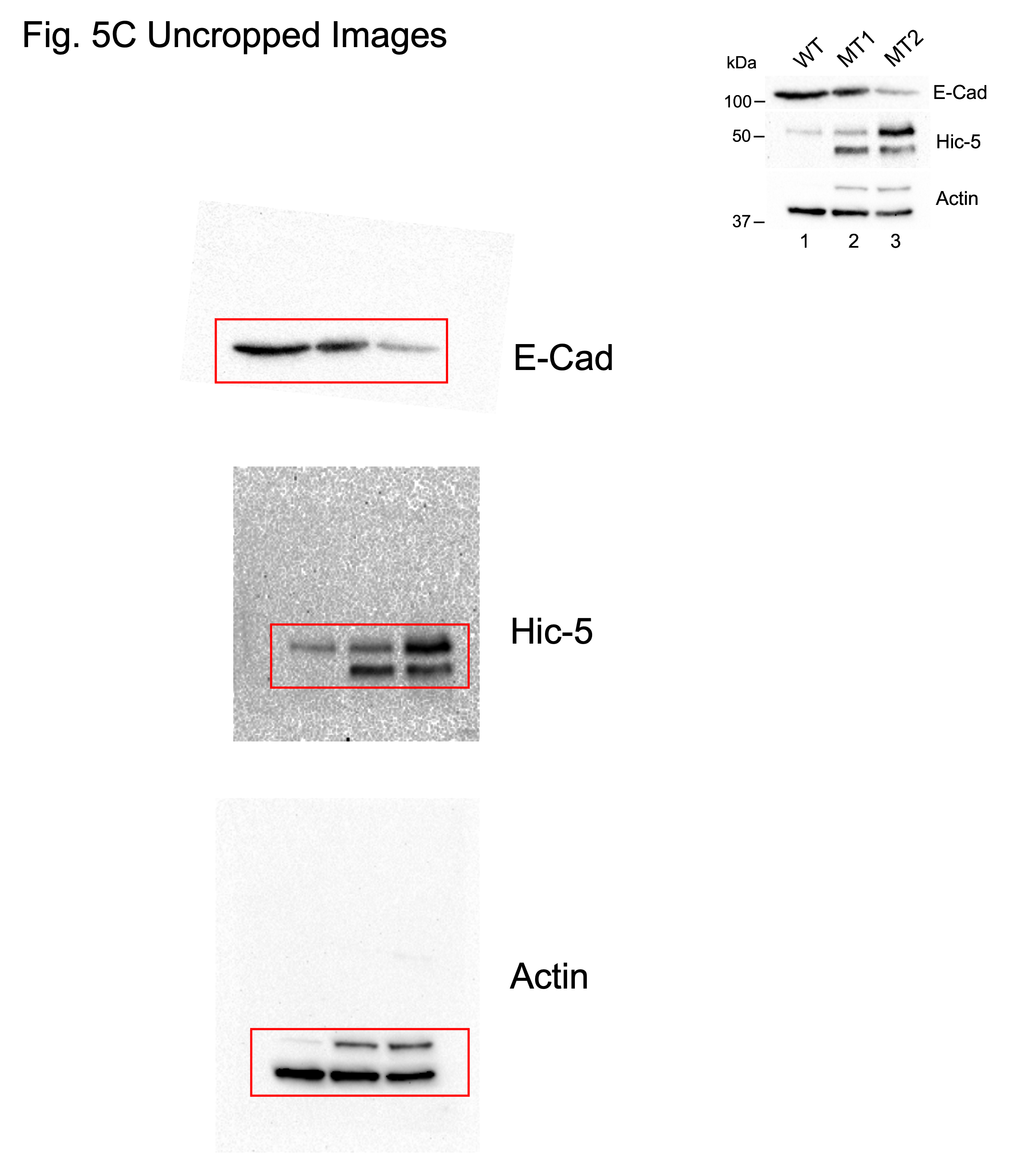


kDa

100-

50-

37-


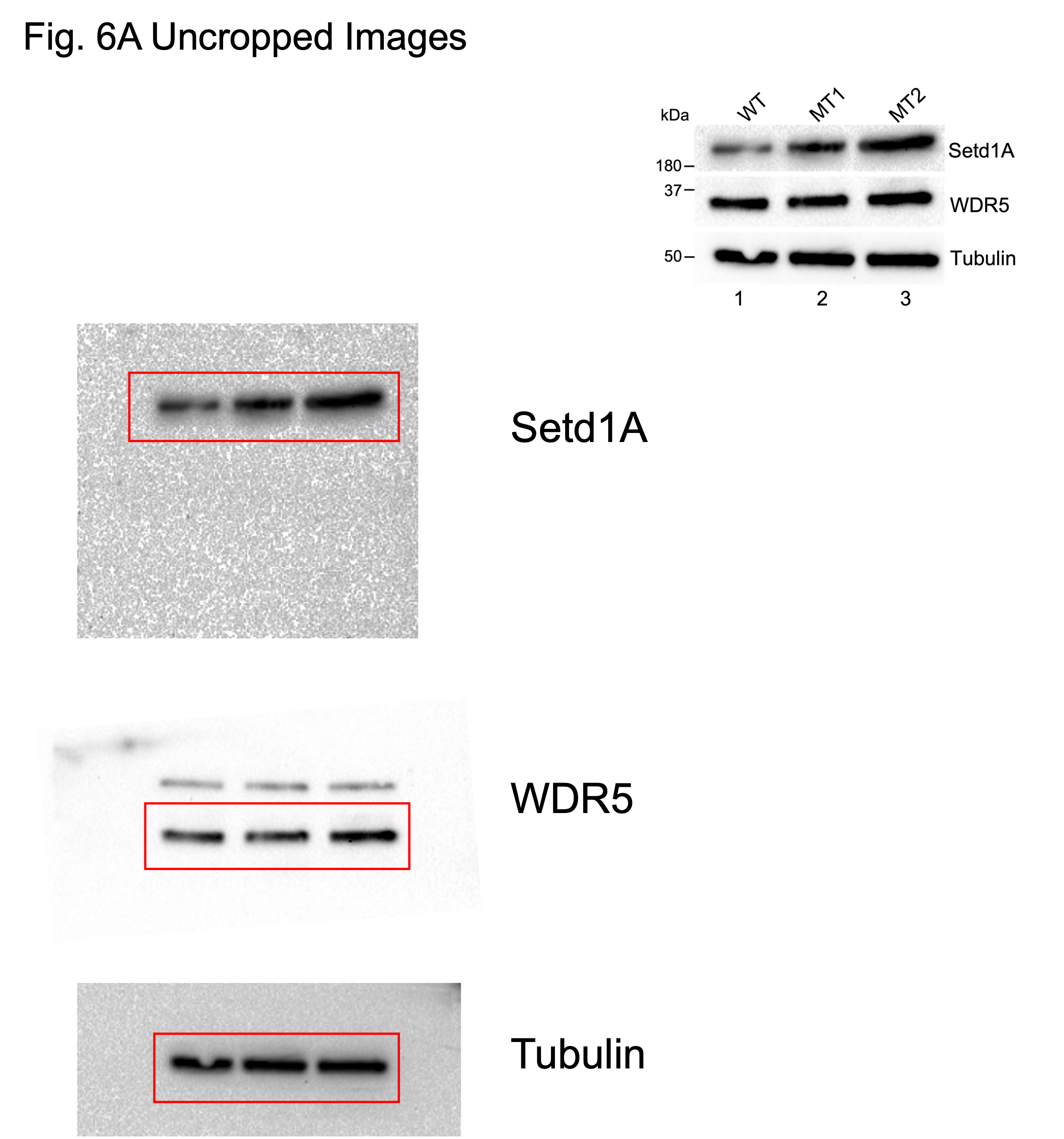


kDa

180-

37-

50-

37-


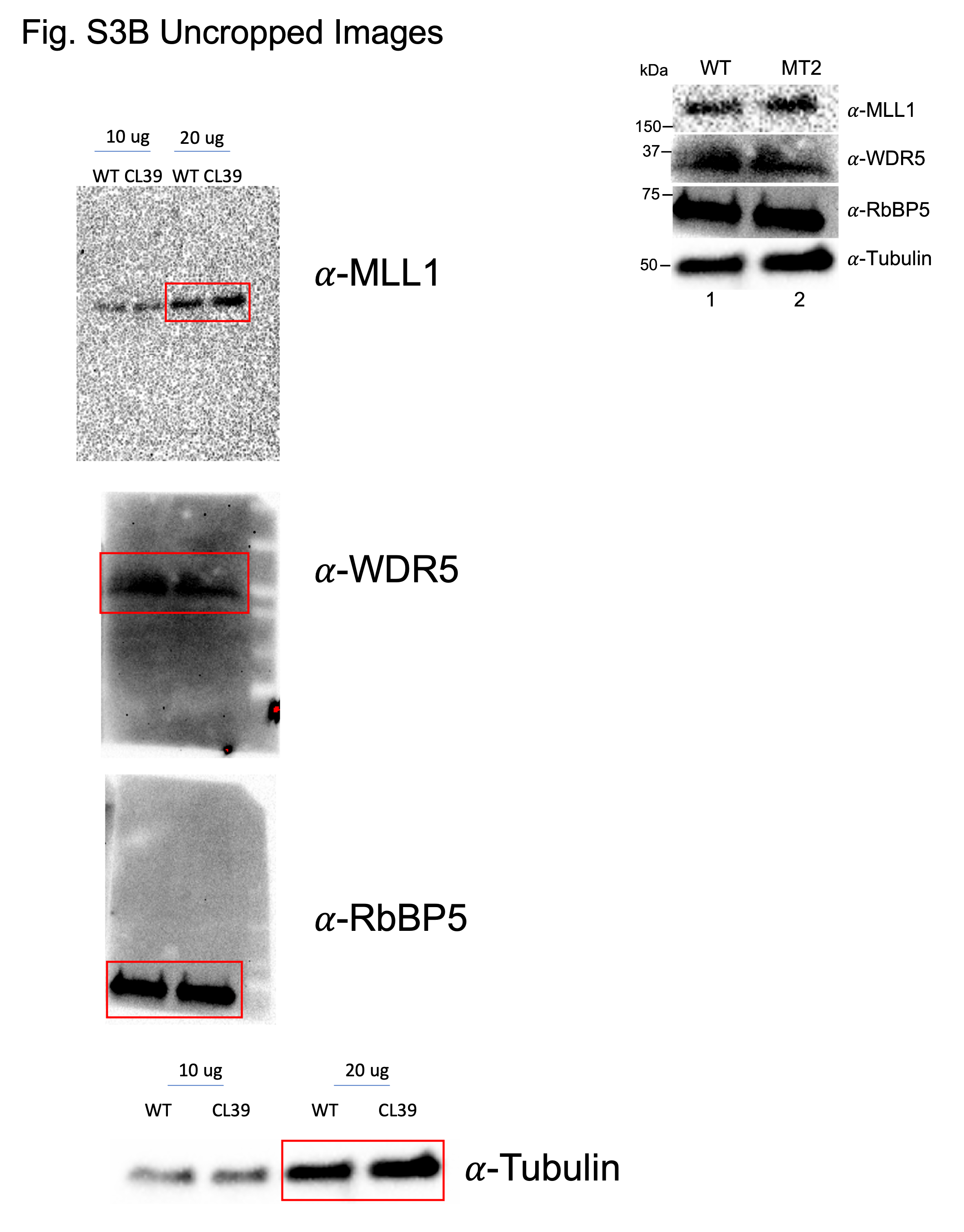


Fig. S4B Uncropped Images

75-

50-

kDa

150-

37-

**Supporting Information**

**Antibodies and Primers**

| **Primary Antibodies** | **Manufacturer, Catalog Number** | **Dilution Factor/Application** |
| --- | --- | --- |
| Mouse anti-E-cadherin | BD Biosciences, 610182 | 1:1000 WB |
| Rabbit anti-E-cadherin | Cell Signaling, 3195 | 1:100 IF |
| Mouse anti-Hic-5 | BD Biosciences, 611165 | 1:1000 WB, 1:100 IF |
| Rhodamine-phalloidin (1:500, R415; Cat# PHDR1, Denver, CO) | Cytoskeleton, R415 PHDR1 | 1:500 IF |
| Rabbit anti-Histone H3 | Millipore, 06-755 | 1:2000 WB |
| Rabbit anti-H3K4me1 | Abcam, ab8895 | 1:1000 WB |
| Rabbit anti-H3K4me2 | Abcam, ab32356 | 1:5000 WB |
| Rabbit anti-H3K4 trimethylation | Active Motif, 39159 | 1:1000 WB |
| Rabbit anti-H3K4 trimethylation ChIP Grade | Epicypher, 13-0041 | 1 ug per ChIP |
| Rabbit anti-Setd1A ChIP Grade | Abcam, ab70378 | 1:2000 WB, 1 ug per ChIP sample |
| Rabbit anti- beta Actin | Abcam, ab8227 | 1:1000 WB |
| Mouse anti-Tubulin | Sigma, T5168 | 1:1000 WB |
| Rabbit anti-WDR5 | Abcam, ab22512 | 1:1000 WB |
| Mouse monoclonal anti-MLL1 | Millipore, 05-765 | 1:1000 WB |
| Rabbit anti-RbBP5 | Bethyl Labs, A300-109A | 1:1000 WB |
| Rabbit IgG negative control  antibody for ChIP | Thermo Fisher Scientific, 31235 | 1 ug per ChIP sample |
| **Secondary Antibodies** |  |  |
| Goat anti-mouse DyLight 488-conjugated | Thermo Fisher, 35502 | 1:250 IF |
| Goat anti-mouse DyLight 550-conjugated | Thermo Fisher, 84540 | 1:250 IF |
| Goat anti-rabbit DyLight 488-conjugated | Thermo Fisher, 35552 | 1:250 IF |
| Goat anti-rabbit DyLight 550-conjugated | Thermo Fisher, 84541 | 1:250 IF |
| Secondary anti-rabbit IgG, HRP-conjugated | GE, NA934VS | 1:30,000 |
| Secondary anti-mouse IgG, HRP-conjugated | GE, NA931VS | 1:10,000 |

| **Primer Name** | **Forward Primer (5’>3’)** | **Reverse Primer (5’>3’)** |
| --- | --- | --- |
| **ChIP-qPCR** |  |  |
| ZFP42 | ATTGGGCATTCCAGCCTACC | GAGTTCAGCTCCTTGGACCC |
| ACTIN | AGCCTCGCCTTTGCCGA | GCGCGGCGATATCATCATC |
| MMP1 | ATGCTGAAACCCTGAAGGTG | CTGCTTGACCCTCAGAGACC |
| MEIS1 | TGTGTAAGACGCGACCTGTT | CCGTGCGTGTGTAAAGTGTG |
| GAPDH | TACTAGCGGTTTTACGGGCG | TCGAACAGGAGGAGCAGAGAGCGA |
| HOXA9 | AGTGGTCTCACTTCCTCCCA | GCTGAGCAGTAAAAGGTGGC |
| **RT-qPCR** |  |  |
| DLK1 | GGGCACAGGAGCATTCATAG | GACGGGGAGCTCTGTGATAG |
| TBXT | GCCCTCTCCCTCCCCTCCACGCACAG | CGGCGCCGTTGCTCACAGACCACAGG |
| SOX17 | CGCTTTCATGGTGTGGGCTAAGGACG | TAGTTGGGGTGGTCCTGCATGTGCTG |
| GAPDH | ACATCGCTCAGACACCATG | TGTAGTTGAGGTCAATGAAGGG |

**References**

1 Alicea-Velazquez, N. L. *et al.* Targeted Disruption of the Interaction between WD-40 Repeat Protein 5 (WDR5) and Mixed Lineage Leukemia (MLL)/SET1 Family Proteins Specifically Inhibits MLL1 and SETd1A Methyltransferase Complexes. *J Biol Chem* **291**, 22357-22372 (2016). <https://doi.org:10.1074/jbc.M116.752626>

2 Dharmarajan, V., Lee, J. H., Patel, A., Skalnik, D. G. & Cosgrove, M. S. Structural basis for WDR5 interaction (Win) motif recognition in human SET1 family histone methyltransferases. *J Biol Chem* **287**, 27275-27289 (2012). <https://doi.org:10.1074/jbc.M112.364125>

3 Patel, A., Vought, V. E., Dharmarajan, V. & Cosgrove, M. S. A conserved arginine-containing motif crucial for the assembly and enzymatic activity of the mixed lineage leukemia protein-1 core complex. *J Biol Chem* **283**, 32162-32175 (2008). <https://doi.org:10.1074/jbc.M806317200>

4 Patel, A., Vought, V. E., Dharmarajan, V. & Cosgrove, M. S. A novel non-SET domain multi-subunit methyltransferase required for sequential nucleosomal histone H3 methylation by the mixed lineage leukemia protein-1 (MLL1) core complex. *J Biol Chem* **286**, 3359-3369 (2011).

5 Shinsky, S. A., Monteith, K. E., Viggiano, S. & Cosgrove, M. S. Biochemical reconstitution and phylogenetic comparison of human SET1 family core complexes involved in histone methylation. *J Biol Chem* **290**, 6361-6375 (2015). <https://doi.org:10.1074/jbc.M114.627646>
